# Collective action against corruption in Western and non-Western countries: cross-cultural implications of the Axiological-Identitary Collective Action Model

**DOI:** 10.3389/fpsyg.2024.1269552

**Published:** 2024-03-20

**Authors:** Dmitry Grigoryev, Albina Gallyamova, Lucian Gideon Conway, Alivia Zubrod, José Manuel Sabucedo, Marcos Dono, Anastasia Batkhina, Klaus Boehnke

**Affiliations:** ^1^HSE University, Moscow, Russia; ^2^Grove City College, Grove City, PA, United States; ^3^Park University, Parkville, MO, United States; ^4^University of Santiago de Compostela, Santiago de Compostela, Spain; ^5^Constructor University, Bremen, Germany

**Keywords:** collective action, system justification, perceived efficacy, national identification, moral obligation, protest against political corruption

## Abstract

People sometimes protest government corruption, yet our current understanding of why they do so is culturally constrained. Can we separate *pancultural* factors influencing people’s willingness to protest government corruption from factors *culturally specific* to each socioecological context? Surprisingly little cross-cultural data exist on this important question. To fill this gap, we performed a cross-cultural test of the Axiological-Identitary Collective Action Model (AICAM) regarding the intention to protest against corruption. As a collective action framework, AICAM integrates three classical antecedents of collective action (injustice, efficacy, identity) with axiological variables (ideology and morality). A total sample of 2,316 participants from six countries (Nigeria, Russia, India, Spain, United States, Germany) in a multilevel analysis of AICAM predictions showed that the positive relationship of the intention to protest corruption with moral obligation, system-based anger, and national identification can be considered pancultural. In contrast, the relationships between system justification and perceived efficacy are culturally specific. System justification negatively predicted the intention to participate only in countries with high levels of wealth, while perceived efficacy positively predicted it only in countries perceived as less corrupt. These findings highlight the importance of accounting features of socioecology and separating pancultural from culture-specific effects in understanding collective action.

## Introduction

People often worry or suffer from common issues that compel them to engage in collective action to achieve specific goals. Political corruption can be considered such a problem: People might attempt to address political corruption through collective action. It is noteworthy that corruption existed in most ancient city-states and continues to be present in modern democratic states today. According to the widely used definition proposed by Transparency International, *corruption* is the abuse of entrusted power for personal gain. Corruption can be considered as an illegal behavior or crime (i.e., ‘illegal use of power’). As such, it generally involves the violation of social norms by, among others, being dishonest (see, e.g., Vilanova et al., 2022). In turn, corruption negatively affects almost all spheres of society and therefore has serious political and socio-economic consequences (from disruption of the mechanism of market competition and unfair redistribution of vital goods to a decrease in trust in society). In this work, we focus on *political* forms of corruption: The abuse of entrusted power by politicians and that may be attributed to both politicians and the political system itself by citizens (Amundsen, 2006).

Hopes to fight corruption are often pinned on civil society through the so-called strengthening democratic accountability of political institutes or democratic practices such as voting. However, attempts to reduce or contain corruption through these mechanisms often fail. Although in theory voters may ‘punish’ corruption by voting against those perceived to be corrupt, empirical research shows that political corruption reduces voting behavior in general (Caillier, 2010). Indeed, part of the reason corruptions persists is that citizens often refuse to use their right to participate in collective action against corruption, which can lead to the persistence of even deeply corrupt systems (Bauhr, 2017). For instance, cross-national comparative research demonstrates that less than half of citizens engaged in protest within the five past years (Kwak, 2022).

What differentiates those who protest political corruption from those who do not? In the present project, across different national settings, we aim to better understand how the drivers of collective action against political corruption works.

### Conceptualization of collective action

The role of corruption in political engagement is complicated, presenting a notable paradox. On the one hand, it increases the level of protest activity approval. On the other hand, it leads to voter disappointment discouraging people from actually *taking* action in group contexts (Kostadinova, 2009; Školník, 2022). Given this complexity, it is important to directly address what we mean by the term *collective action*. At a broad level, collective action involves a behavior that is done by an individual jointly with other people as *representatives* of a group (Wright et al., 1990). This implies that any person performing an individual action as a representative of their group with group goals in mind could be involved in collective action (van Zomeren and Iyer, 2009). Nevertheless, these ‘individual’ collective actions (such as protest voting) have been defined separately from other collective behaviors as they may have different determinants (Otjes et al., 2020). In this work, we conceptualize collective action as involvement in clear protest behaviors (Olson, 1971; Lichbach, 1994).

We do not consider behaviors such as voting in our work due to their ambiguous conceptual relationship to collective action targeted at political corruption. Voting behaviors are multifaceted and thus not always clearly related to collective actions aimed at widespread, systemic corruption. Further, it may be a false assumption (as in some of the countries in the present work) that citizens perceive the democratic voting system as fully functioning. As a result, it is more scientifically appropriate to focus on targeted protests as outcomes for collective actions related to political corruption. Our investigation employs the Axiological-Identitary Collective Action Model (AICAM; see Sabucedo et al., 2019) to examine the intention to participate in anti-corruption protests in varying levels of wealth and perceived corruption in six countries (Nigeria, Russia, India, Spain, United States, Germany).^1^ Since attitudes toward corruption as all attitudes regarding injustices and dishonesty have moral roots, we believe that AICAM can be especially useful for this scope. We now turn our attention to this model.

### AICAM: understanding the call to collective action

Sociologist Gamson (1992) identified three factors that facilitate, justify, and legitimize collective action: injustice, efficacy, and identity. Subsequently, these factors formed the basis of the Social Identity Model of Collective Action (SIMCA), which received meta-analytical support (see van Zomeren et al., 2008). As suggested by this model, if relative deprivation is the cognitive aspect of perceived injustice, then *anger* is its emotional side (so-called affective injustice), which is according to this meta-analysis better than the cognitive side in predicting participation in collective action. Another basic condition for collective action is *perceived efficacy:* an assessment of whether collective action will allow people to move toward their goal. Finally, *social identity* (and in particular politicized identity) is the component without which no collective action is possible. Social identity strengthens the perception of injustice and belief in the ability of the group to achieve change. In contrast, the Encapsulation Model of the Social Identity of Collective Action (EMSICA), developed as a variation of the SIMCA model, suggested that emotions of moral outrage shape new social identity that in the combination with collective efficacy beliefs may be led by social injustice. In other words, EMSICA considers social identity not in the form of a pre-existing group membership but forming by current shared beliefs and views (Thomas et al., 2012).

AICAM does not abandon these validated classical antecedents of collective action but suggests a more comprehensive picture, which includes both perceived factors (perceived injustice, perceived efficacy) and internal motives (ideology, moral obligation, social identity). Indeed, AICAM combines utilitarian and moral explanations for collective action and suggests an explanation of the socio-psychological mechanism of collective action that, in general, is not so much connected with the evaluation of reality but more rooted deeply in mindsets (e.g., values). This enhancement improves the simpler previous explanation, which suggests: (1) protesters must identify with a social group, (2) be aware that their group has suffered injustice, and (3) believe that their collective efforts can lead to social change. Although such findings doubtlessly are of predictive value, an understanding of the consequences of ideological factors is clearly lacking (Jost et al., 2017). As people do not always rationally weigh all consequences, it is crucial to include factors predicting why people engage even in likely unsuccessful collective action despite the expectable costs.

### Advantages of the axiological-identitary path

The point is that *ideology* guides the interpretation of a given situation, providing criteria for evaluating what is (un)fair and who should be blamed. In addition, taking ideology and the values it offers into account in the context of collective action also requires considering the will power to act in accordance with the goals that the ideology deems to be fair and right. That is, it is important to take into account *moral obligation* to act according to what you believe in, thereby overcoming the costs associated with achieving the goals set (Sabucedo et al., 2019). This may be especially relevant for the motivation to participate in protests in non-democratic societies, due to the risk of reprisals (Ayanian et al., 2021). Thus, another advantage of AICAM is that the model differentiates beliefs or convictions from the moral obligation to act in accordance with them. The level of feeling obliged will vary between people who may hold the same beliefs and situations. For a truly comprehensive understanding of the motivations of protesters, it is important not only to understand who they are (*their identity*), but also what they stand for, as well as how far they are willing to go to achieve their goal (*their axiology*; Jost et al., 2017; Sabucedo et al., 2019; Agostini and van Zomeren, 2021).

#### Ideology

In previous testing of AICAM, the ideology construct was operationalized as ideological self-placement (left or right). Of course, ideology is more complicated than “left or right,” but ideological self-placement reflects the main aspects of ideology (political values, party identification, and political attitudes and opinions; Sabucedo et al., 2019). In this study, we decided to consider *system justification* instead of ideological self-placement. Collective action itself can be defined as any joint effort to challenge or maintain the status quo regardless of their group status (Solak et al., 2021). At the same time, in the most general terms, according to System Justification Theory, people are motivated to maintain the status quo, i.e., system justification is to defend, justify, accept, rationalize, and support the social, political, and economic systems in which they live and work (Jost et al., 2017). This motivation is rooted in (1) the existential need to reduce threat and anxiety; (2) the epistemic need to see social reality as coherent, structured, and ordered, and (3) the relational need to see it in harmony with other people (Jost, 2015; Friesen et al., 2019). An important consequence of system justification is the so-called ‘palliative effect’ when system justification (1) increases subjective well-being, positive affect, life satisfaction, subjective sense of security and (2) reduces cognitive dissonance, moral outrage, anger, frustration, helplessness (Solak et al., 2021).

Considering system justification also can be useful because it does not involve any certain political views, but rather just measures attitudes toward current system. This is important because ideological placement does not play the same role in all societies. We suggest that in non-democratic states the potential for collective actions such as protest may be predicted more precisely by assessing how individuals justify the system, as there may not be defined political beliefs in countries where a political pluralistic system does not exist. Moreover, recent studies found that both left- and right-wing authoritarianism strongly connect with attitudes related to bolstering the status-quo such as dogmatism and prejudice (Conway et al., 2020). Therefore, at least for our aim, system justification may be more helpful for predicting collective action: System justification, by restraining the awakening of the dynamics of a sense of injustice (see Gaucher and Jost, 2011), can act as a key factor undermining the protest against unfair social phenomena, such as corruption. Indeed, a negative relationship has already been found between system justification and the perception of nation-level corruption, which was explained by the fact that, to some extent, corruption can be perceived by people as not being a threat (see Tan et al., 2016).

Importantly, System Justification Theory also suggests that believing a system is just may also make it *more* likely for people to protest. There is reason to believe that system justification is positively related to perceived efficacy, since a certain level of faith in the system is necessary to believe that the system will respond to individual efforts to “reform it from within,” and this should encourage rather than discourage political activity (Cichocka and Jost, 2014). This relationship can be quite stable. It has been shown that system justification is positively associated with perceived efficacy in both high-status and low-status groups (Osborne et al., 2019). In addition, system-based emotions should predict whether collective action is likely and what form it will take; decreased anger at the system can mediate the relationship between system justification and collective action (Jost et al., 2017). Finally, in a sense, a moral obligation is a consequence of the very existence of a certain belief system (Sabucedo et al., 2019; Dono et al., 2021), and since system justification refers to the achievement of desired goals, it is reasonable to assume that it reduces the intention to act for their achievement, i.e., prevents the actualization of the moral obligation. On the other hand, as argued above, it is important from the point of view of System Justification Theory to recognize that the decision to participate in the protest is an inherently ideological decision, since it includes, among other things, a critical assessment of the (il)legitimacy of the existing regime (Badaan et al., 2018). Thus, system justification can undermine the intention to protest even among political activists with a firmly shaped politicized identity (Jost et al., 2017).

#### Identity

According to AICAM, ideology and identity are distal antecedents of collective action, which are related to each other bidirectionally, since group identity becomes more rooted due to sharing attitudes and values, whereas ideology is strengthened by the acting of groups maintaining certain ideas (Sabucedo et al., 2019). Similarly, like utilizing system justification instead of ideological self-placement, we decided to use *national identification* instead of other politicized identities, because it may better reflect attitudes toward corruption in most countries. Indeed, in the most general terms, national identity describes belonging to a political community that has institutions, rights, and obligations for all its members (Milfont et al., 2020), which may be significant for people to consider the fight against corruption in their country as part of their national identity. Thus, to the extent that national identification is central and meaningful to people, their intention to engage in collective action can represent a significant effort to strengthen the position of their nation (Stathi et al., 2019), especially considering that political corruption affects the whole society, not just any particular group.

Moreover, national identification can be deeply involved in all other factors of the intention to participate, since relevant social identity is the psychological basis for the implementation of any collective action (van Zomeren et al., 2011). Also, national identification can act as a predictor of justifying belief systems (Vargas-Salfate et al., 2018). Thus, while the role of system justification in relation to the perceived efficacy in the intention to protest against corruption in a country is more complex, a more or less straightforward overall picture emerges for the role of national identification.

### Current study

**Figure 1:**
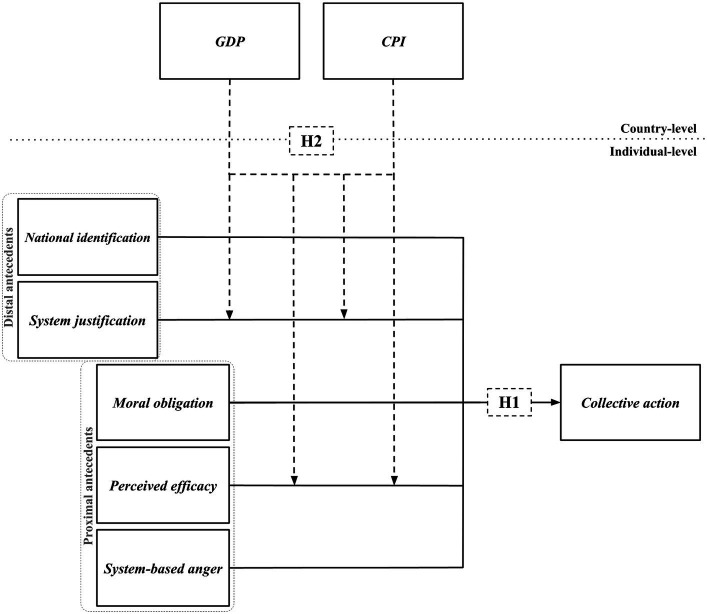
The suggested conceptual model.

In this study, we cross-culturally test the AICAM predictions regarding collective action against corruption. First, AICAM proposes two distal (national identification, system justification) and three proximal (system-based anger, moral obligation, perceived efficacy) antecedents of the intention to participate in protests against corruption. Our scope is to understand how this model works without being tied to certain political views (left or right), so we replaced the variable ideology with a system-level factor. Hence, we suggest a framework that involves epistemic, existential, and relational needs incorporating condemnation or affirmation of the political status quo. The suggested conceptual model is displayed on Figure 1.

According to the previous literature, all components of the model should be positively related to collective action, except for system justification, which should show a negative relationship (H1). In this study, we aim to test to what extent these predictions hold in different countries with varying level of wealth and corruption, since these pancultural expectations may not take into account the socio-cultural contexts of different societies. Some differences between countries previously have been found. For example, national identification is higher in less developed countries, whereas system justification is higher in more developed countries (Vargas-Salfate et al., 2018; see also Caricati and Lorenzi-Cioldi, 2012). It is not entirely clear what the implications of this might be for participating in protests. Second, what are the implications of the interplay of country characteristics and individual differences in motivation regarding the intention to engage in collective action? These elements could explain the peculiarities of the role of system justification and perceived efficacy in collective action, as mentioned above.

Regarding country characteristics and AICAM, we were interested in the wealth of the country and the level of perceived corruption, which are reflected in the level of GDP *per capita* (GDP) and the Corruption Perceptions Index (CPI). First, rich countries have more developed democratic institutions that can keep levels of corruption in check (e.g., Inglehart and Welzel, 2005).^2^ Second, a country’s wealth strengthens system justification (see Caricati and Lorenzi-Cioldi, 2012). Finally, cross-level interaction has shown that system justification is negatively related to collective action in more individualistic countries only (see De Cristofaro et al., 2022), whereas individualism is known to be positively associated with the wealth of the country and the level of democracy (e.g., Hofstede, 2001). In addition, it is assumed that the CPI score of countries may arise from a cycle of self-fulfilling prophecies regarding opportunities for economic development, and itself significantly affects the perception of corruption by their inhabitants (Warren and Laufer, 2009). In other words, if a country emerges as having a high CPI score (which stands for low corruption) it will subsequently be seen as less corrupted which in turn will cause greater economic development and vice versa. Also, for protesters who are subject to repression (that is more common in non-democratic societies), perceived efficacy may not be the typical driver to protest (Ayanian et al., 2021).

Thus, we expected that these nations’ characteristics interact with system justification and perceived efficacy in such a way that system justification negatively predicts the intention to participate in the protest, whereas perceived efficacy positively predicts it but only in rich countries (H2). That is, the motivation to justify the system can only be observed in rich and democratic countries, when it is possible to believe in the strength and effectiveness of institutions that counteract corruption, the same belief can be relevant for the perceived efficacy that, if necessary, inhabitants of rich democracies can easily achieve their goal. In addition, both the negative association for system justification and the positive association for perceived efficacy in the overall model will be weakened by the level of perceived corruption in the country (H3),^3^ since the perception of the inhabitants of their country as deeply corrupt can undermine both faith in the legitimacy of the system in general, and faith in their own strength to change something. However, they may be comforted by the belief in a just world, i.e., the expectation that, since the world is just, everything will turn out well in the long run and, therefore, no immediate action is required to eliminate the injustice of the system (see Stroebe, 2013). The current empirical study tested these hypotheses in the context of six countries that differ quite widely in terms of wealth and levels of perceived corruption. Doing so allowed us to importantly tease apart pancultural effects from culture-specific effects.

## Method

### Participants

The total sample consisted of 2,316 participants from six countries (Nigeria, Russia, India, Spain, United States, Germany), among which there were 44% women and 56% men aged 16 to 75 (*M* = 29.9, *SD* = 10.0); 39% of them were students. A description of participants by country is available in Table 1.

**Table 1:** Description of the samples from six countries (*N* = 2,316).

	Year	Language	CPI	GDP	*N*	% of men	*M_age_* (*SD*), range	% of students
Nigeria	2019	English	26	2229.9	247	73	29.6 (7.7), 18–59	41
Russia	2018	Russian	28	11287.4	402	42	26.8 (9.4), 16–65	39
India	2019	English	41	2100.8	417	82	28.1 (7.5), 17–64	37
Spain	2018	Spanish	58	30349.8	441	51	31.5 (10.0), 17–69	41
USA	2018	English	71	63064.4	409	49	35.1 (12.0), 18–71	29
Germany	2018	German	80	47950.2	400	45	28.2 (9.8), 18–75	48

CPI, Corruption Perceptions Index; GDP, GDP *per capita*, current US$.

### Procedure

Data was collected online in 2018 (for Russia, Spain, USA, Germany) and 2019 (for Nigeria, India) using the Amazon MTurk platform (for Spain, USA) and Clickworker (for Nigeria, India, Russia, Germany). Such platforms with crowdsourced subject pools are recognized as suitable for conducting cross-cultural research (see, e.g., Paolacci et al., 2010; Aguinis et al., 2021). Participants were remunerated approximately 1 US$ for completing the questionnaire; they had to fill out a questionnaire and read the instructions, which included basic information about the research problem, information about confidentiality, as well as contact details of the researchers.

#### Power analysis

To determine the required sample size for linear mixed models, a statistical power analysis was performed in R using the sjstats package (Lüdecke et al., 2021). This study focused on an effect size of *r* = 0.10, which according to Cohen’s cutoffs refers to a small effect size, with alpha = 0.05 and power = 0.80 recommended. In total, the minimum required number of recruited participants, according to the results of the calculations, was 787 people, 131 people per country.

### Measures

The questionnaire was presented to participants from each country in the respective state language (see Table 1). The measures that had not previously been translated from English into Russian, Spanish, and German were translated and adapted by native speakers and tested in subsequent statistical analyses to determine reliability (internal consistency) and factor structure. Moreover, we tested measurement invariance with the alignment method, when effect sizes of approximate invariance include a sufficiently high *R^2^* value this indicates a high degree of measurement invariance (see Asparouhov and Muthén, 2014). Average *R^2^* values for loadings and intercepts in the results were higher than 0.90, showing that the measurement model had a sufficient level of metric and scalar invariance for our cross-cultural comparisons. In addition, each questionnaire also contained questions about sociodemographic characteristics (gender, age, student status). All measures had a 7-point Likert scale for response (from 1 = *strongly disagree* to 7 = *strongly agree*).

#### Dependent variable

##### Collective action

Two items were aimed at assessing the intention to participate in anti-corruption protests (α_Nigeria_ = 0.82; α_Russia_ = 0.91; α_India_ = 0.79; α_Spain_ = 0.81; α_USA_ = 0.86; α_Germany_ = 0.83; adapted from Tausch et al., 2011). Example: “I am ready to support protests (e.g., rallies, marches) against corruption in […]” (*M*_Nigeria_ [*SD*] = 5.70 [1.47]; *M*_Russia_ [*SD*] = 4.54 [1.77]; *M*_India_ [*SD*] = 5.66 [1.47]; *M*_Spain_ [*SD*] = 5.47 [1.40]; *M*_USA_ [*SD*] = 4.41 [1.71]; *M*_Germany_ [*SD*] = 4.13 [1.49]).

#### Independent variables

##### National identification

Two items were aimed at assessing national identification (α_Nigeria_ = 0.79; α_Russia_ = 0.74; α_India_ = 0.86; α_Spain_ = 0.82; α_USA_ = 0.92; α_Germany_ = 0.88; Verkuyten and Yildiz, 2007; Grigoryev et al., 2022). Example: “I feel like a part of […] society” (*M*_Nigeria_ [*SD*] = 6.25 [1.28]; *M*_Russia_ [*SD*] = 4.84 [1.55]; *M*_India_ [*SD*] = 6.31 [1.41]; *M*_Spain_ [*SD*] = 5.74 [1.51]; *M*_USA_ [*SD*] = 5.46 [2.01]; *M*_Germany_ [*SD*] = 4.29 [1.52]).

##### System justification

Eight items were aimed at assessing system justification (ω_Nigeria_ = 0.83; ω_Russia_ = 0.90; ω_India_ = 0.81; ω_Spain_ = 0.86; ω_USA_ = 0.92; ω_Germany_ = 0.89; Ullrich and Cohrs, 2007; Jost, 2015; Grigoryev et al., 2022). Example: “Most policies serve the greater good” (*M*_Nigeria_ [*SD*] = 3.63 [0.97]; *M*_Russia_ [*SD*] = 2.46 [1.15]; *M*_India_ [*SD*] = 4.35 [1.19]; *M*_Spain_ [*SD*] = 3.27 [0.98]; *M*_USA_ [*SD*] = 4.08 [1.18]; *M*_Germany_ [*SD*] = 3.85 [1.04]).

##### Moral obligation

Five items were aimed at assessing moral obligation (ω_Nigeria_ = 0.84; ω_Russia_ = 0.92; ω_India_ = 0.89; ω_Spain_ = 0.92; ω_USA_ = 0.91; ω_Germany_ = 0.92; Sabucedo et al., 2018). Example: “To mobilize against corruption in […] constitutes a moral obligation to oneself” (*M*_Nigeria_ [*SD*] = 5.42 [1.18]; *M*_Russia_ [*SD*] = 4.11 [1.54]; *M*_India_ [*SD*] = 5.33 [1.19]; *M*_Spain_ [*SD*] = 4.69 [1.46]; *M*_USA_ [*SD*] = 4.36 [1.39]; *M*_Germany_ [*SD*] = 3.71 [1.31]).

##### Perceived efficacy

Two items were aimed at assessing perceived efficacy (α_Nigeria_ = 0.74; α_Russia_ = 0.80; α_India_ = 0.84; α_Spain_ = 0.88; α_USA_ = 0.86; α_Germany_ = 0.86; adapted from Tausch et al., 2011; Shuman et al., 2016). Example: “I can contribute to the collective actions that affect society as a whole” (*M*_Nigeria_ [*SD*] = 5.14 [1.53]; *M*_Russia_ [*SD*] = 4.02 [1.53]; *M*_India_ [*SD*] = 5.05 [1.52]; *M*_Spain_ [*SD*] = 4.46 [1.54]; *M*_USA_ [*SD*] = 4.50 [1.40]; *M*_Germany_ [*SD*] = 5.69 [1.46]).

##### System-based anger

Two items were aimed at assessing system-based anger (α_Nigeria_ = 0.87; α_Russia_ = 0.92; α_India_ = 0.85; α_Spain_ = 0.81; α_USA_ = 0.91; α_Germany_ = 0.82; adapted from Tausch et al., 2011; Jost et al., 2012). Example: “I feel anger when I think about the current state of affairs in […]” (*M*_Nigeria_ [*SD*] = 5.75 [1.55]; *M*_Russia_ [*SD*] = 4.79 [1.86]; *M*_India_ [*SD*] = 4.98 [1.78]; *M*_Spain_ [*SD*] = 5.26 [1.41]; *M*_USA_ [*SD*] = 4.55 [1.72]; *M*_Germany_ [*SD*] = 3.76 [1.26]).

#### Additional variables

##### GDP

Estimated Gross Domestic Product *per capita* in current US dollars was retrieved from World Bank for the corresponding year.^4^

##### CPI

Corruption Perceptions Index (from 0 = *the highest level of corruption* to 100 = *the lowest level of corruption*), compiled on the basis of expert assessments and public opinion polls about the perception of the level of corruption in the public sector, was retrieved from Transparency International for the corresponding year.^5^

### Data analysis

To analyze the relationships between individual-level variables, we first conducted a multilevel correlation analysis. Subsequently, we performed a process known as ipsatization on the means of each country. Ipsatization involves adjusting the data by transforming scores on multiple scales so that the mean of these scores is the same for all countries. This method helps to control for any systematic differences or biases in responding that may occur between different countries, thereby enabling a more accurate cross-country comparison (Fischer, 2004). By implementing ipsatization, we effectively control for these cultural biases and ensure that the observed differences between countries are attributable to genuine variations in the phenomena being measured, rather than artifacts of disparate response styles. After ipsatizing the means, we rescaled them within a range from 0.01 to 1, with 1 representing the maximum and 0.01 representing the minimum. This rescaling helps avoid negative scores and provide a common scale of comparison across different measures. The One-way ANOVA was then utilized to compare these newly adjusted means.

For the final step, we tested our primary hypotheses about cross-level interaction through mixed models. In these models, all individual level independent variables were centered cluster-wise to isolate the effects of individual variables within each country cluster (Enders and Tofighi, 2007), while additional country variables were log-transformed to stabilize variance and normalize the distribution (Osborne, 2002). This comprehensive analysis strategy allowed us to robustly examine the relationships between our variables of interest across different cultural contexts.

## Results

The data contained no missing values or outliers. All the scales had satisfactory indicators of internal consistency, the reliability MacDonald’s ω ranged from 0.81 to 0.92 and Cronbach’s α ranged from 0.74 to 0.92, which generally indicates a sufficient reliability of the measurements. The results of the multilevel correlation analysis of individual level focal variables across the entire sample are available in Table 2. All the relationships supported the AICAM predictions. Overall, the intention to participate in anti-corruption protests was more strongly associated with proximal antecedents (moral obligation [0.66], system-based anger [0.44], perceived efficacy [0.26]) than with distal antecedents (national identification [0.17], system justification [−0.13]). Among the antecedents, moral obligation was positively associated with system-based anger (0.37), perceived efficacy (0.28), and national identification (0.13), as well as weakly negatively with system justification (−0.07). At the same time, system justification was negatively associated with system-based anger (−0.25) and positively with perceived efficacy (0.22), while national identification was positively associated with all the antecedents (from 0.04 to 0.18).

**Table 2:** Results of multilevel correlation analysis of the individual level focal variables (*N* = 2,316, *k* = 6).

	1	2	3	4	5
1. Collective action	–				
2. National identification	0.17	–			
3. System justification	−0.13	0.14	–		
4. Moral obligation	0.66	0.13	−0.07	–	
5. Perceived efficacy	0.26	0.18	0.22	0.28	–
6. System-based anger	0.44	0.04	−0.25	0.37	0.15

All the correlation coefficients are significant at *p* < 0.001, except for the relationship between national identification and system-based anger, *r* = 0.04 [0.01, 0.08], *p* = 0.049.

The results of an ANOVA for relative comparison of the ipsatized mean values of individual level focal variables across samples are available in Table 3. The largest percent of variance in values of centered means were observed for system justification (0.19) and perceived efficacy (0.15). At the same time, the largest mean value of system justification was observed in samples from rich and perceived as less corrupt countries (Germany and United States). The Germany sample stood out from other samples with a wide margin in terms of perceived efficacy.

**Table 3:** Comparison of the ipsatized means of the individual level focal variables across samples (*N =* 2,316).

	Nigeria (*N =* 247)	Russia (*N =* 402)	India (*N =* 417)	Spain (*N =* 441)	USA (*N =* 409)	Germany (*N =* 400)	*F*(df1, df2)	ω^2^
Collective action	0.65 (0.20)_a_	0.66 (0.25)_a_	0.65 (0.20)_a_	0.69 (0.19)_a_	0.58 (0.24)_b_	0.59 (0.21)_b_	17.4(5, 2,310)*	0.03
National identification	0.73 (0.18)_ab_	0.70 (0.22)_b_	0.74 (0.20)_a_	0.73 (0.21)_ab_	0.73 (0.28)_ab_	0.61 (0.21)_c_	20.9(5, 2,310)*	0.04
System justification	0.36 (0.14)_c_	0.37 (0.16)_c_	0.47 (0.17)_b_	0.38 (0.14)_c_	0.53 (0.16)_a_	0.55 (0.15)_a_	112.3(5, 2,310)*	0.19
Moral obligation	0.62 (0.16)_a_	0.60 (0.21)_a_	0.61 (0.17)_a_	0.58 (0.20)_a_	0.57 (0.19)_a_	0.53 (0.18)_b_	10.6(5, 2,310)*	0.02
Perceived efficacy	0.58 (0.21)_b_	0.59 (0.21)_b_	0.57 (0.21)_b_	0.55 (0.21)_b_	0.59 (0.20)_b_	0.80 (0.20)_a_	81.4(5, 2,310)*	0.15
System-based anger	0.66 (0.22)_a_	0.69 (0.26)_a_	0.56 (0.25)_bc_	0.66 (0.20)_a_	0.60 (0.24)_b_	0.53 (0.17)_c_	31.7(5, 2,310)*	0.06

Significant differences at the *p* < 0.05 level, adjusted for Tukey’s multiple comparisons, are denoted by differing Latin letters. *p < 0.001.

The results of mixed models predicting the intention to participate in anti-corruption protests are available in Table 4. Model 1 showed that about 17% of the variance in the intention to participate is predicted by the country where the sample was taken from. Model 2, with the addition of individual level focal variables, showed that about 42% of the variance in the intention to participate is associated with them. The intention to participate can be predicted by male gender, younger age, greater national identification, moral obligation, perceived efficacy and system-based anger, as well as by less system justification. The addition of country level predictors (GDP and CPI) to Model 3 improved this individual level prediction by 6% of the variance, without changing the nature of the predictor associations.

**Table 4:** Results of mixed models for six samples (*N* = 2,316, *k* = 6).

	Model				Null (Step 1)	Individual level predictors (Step 2)	Individual and country level predictors (Step 3)	Cross-level interactions (Step 4)
Individual level				
Intercept	4.985 (0.287)***	4.900 (0.283)***	7.673 (2.055)*	7.673 (2.055)*
Gender (1 = men)		0.119 (0.049)*	0.117 (0.049)*	0.117 (0.049)*
Age		−0.009 (0.003)**	−0.009 (0.003)**	−0.009 (0.003)**
Student (1 = yes)		0.043 (0.053)	0.043 (0.053)	0.044 (0.053)
National identification		0.095 (0.015)***	0.095 (0.015)***	0.097 (0.015)***
System justification		−0.095 (0.023)***	−0.095 (0.023)***	0.132 (0.210)
Moral obligation		0.625 (0.019)***	0.625 (0.019)***	0.618 (0.019)***
Perceived efficacy		0.079 (0.017)***	0.079 (0.017)***	−0.220 (0.147)
System-based anger		0.195 (0.016)***	0.195 (0.016)***	0.193 (0.016)***
Country level				
GDP			−0.434 (0.283)	−0.434 (0.283)
CPI			0.350 (0.896)	0.351 (0.896)
Cross-level interactions				
System justification × GDP				−0.072 (0.025)**
System justification × CPI				0.118 (0.082)
Perceived efficacy × GDP				−0.017 (0.019)
Perceived efficacy × CPI				0.121 (0.059)*
Variance components				
Within-country (L1) variance (σ^2^)	2.440	1.234	1.234	1.228
Intercept (L2) variance (τ_00_)	0.487	0.469	0.317	0.317
Additional information				
ICC	0.17	0.28	0.20	0.21
AIC	8669.4	7103.1	7101.7	7093.4
*R^2^*_m_		0.42	0.48	0.49
*R^2^_c_*	0.17	0.58	0.59	0.59

*p < 0.05; **p < 0.01; ***p < 0.001.

Finally, the subsequent addition of cross-level interactions further improved Model 4, bringing the total share of explained variance in the intention to participate in anti-corruption protests to 59%. The addition of cross-level interactions to the prediction model also showed that, according to a simple slope analysis in this improved model, system justification emerged as a negative predictor of the intention to participate in protest only in countries with high levels of wealth (*B* [95% *CI*] = −0.188 [−0.263, −0.113], *p* < 0.001). At the same time, perceived efficacy positively predicted the intention to participate in protest only in countries perceived as less corrupt (*B* [95% *CI*] = 0.129 [0.072, 0.186], *p* < 0.001).

## Discussion

The current study offered a novel cross-cultural test of AICAM in the context of protest against corruption in various countries. Our study allowed us to test the degree that these predictions were pancultural (across the samples studied) versus culture-specific. The results showed that for the six countries considered, the positive association of the intention to participate with moral obligation, system-based anger, and national identification can be considered pancultural (and attributed to individual differences of the protesters), while the association with the system justification and perceived efficacy is culturally specific (i.e., dependent on interaction with the characteristics of the country context).

### Moderation of socioecological context

System justification negatively predicted the intention to engage in collective action only in countries with high levels of wealth, while perceived efficacy positively predicted the intention to engage in collective action only in countries perceived as less corrupt. These findings are convergent to some of those reported earlier (see Sabucedo et al., 2019; Ayanian et al., 2021; De Cristofaro et al., 2022). They add new insights regarding the role of socioecology in collective action. In wealthier, individualistic societies, a higher system justification seems to deter collective action. This might be explained by a higher need to maintain the status quo in these societies where the system is perceived as more beneficial (De Cristofaro et al., 2022). Moreover, system justification may be associated with lower perceptions of corruption among individuals in these societies, as they may downplay societal flaws in order to defend the current system (Tan et al., 2016). On the other hand, in less corrupt societies, individuals with a high perceived efficacy are more likely to engage in collective action, confident in their potential to effect change. In the more corrupt contexts, such faith might be eroded, impacting citizens’ participation in collective action. In addition, our results add understanding about how national identification and system justification, as another operationalization of identity and ideology within AICAM, predict collective action. Thus, this research (1) allowed us to incorporate socioecological perspectives in the theoretical framework of collective action; (2) enriched our understanding about how national identification and system justification predict collective action against corruption; and (3) generated new conclusions via revision of AICAM – specifically, adding another operationalization of identity and ideology.

Our results highlight that system justification is sensitive to specific country context. Social change is more likely to be accepted by citizens of a given country when it is sanctioned by the system and therefore imbued with the legitimacy of the overarching social system (Gaucher and Jost, 2011). However, some types of societies (e.g., post-communist) are characterized by lower levels of system justification (see Cichocka and Jost, 2014). When the current system fails to satisfy existential, epistemic, and relational needs, people in such societies may take some comfort in perceiving it as predictably malicious and unjust. Low levels of system justification seem to be associated with the perception of the system as sanctioning in a completely random or meaningless manner. When this is accompanied by subjective states that are symptoms of social anomie and political alienation, it suggests that existential, epistemic and relational needs are completely frustrated. Perceived efficacy and protests against corruption in democratic societies with a high level of system justification, at the same time, seem to foster the so-called ‘free rider effect’, where the majority of the population will just expect other fellow citizens to fight corruption effectively. A non-negligible percentage of this majority may be even less willing to face the costs of collective action (Bauhr, 2017).

However, perceived efficacy is positively related with the intention to engage in collective action only in countries that are perceived to be less corrupt. This finding is especially important given that belief in the potential for a protest movement to effect change is a crucial factor legitimizing social protests in the eyes of the non-participating majority, who, despite not participating, are affected by the same social issues (Jiménez-Moya et al., 2019). Indeed, we assume that when (most) non-activists agree with and legitimize social protest, they can act as passive supporters who further the protesters’ goals (e.g., by voting for a political party that will take into account the demands of society, or by influencing attitudes of politicians to a certain social problem). In contrast to the characteristics of collective action in democratic countries, protesters within repressive undemocratic societies are not driven by political efficacy in the first place (Ayanian et al., 2021). Indeed, as noted earlier, in those cases the moral obligation may be more significant, which emphasizes that people can participate in collective actions regardless of their effectiveness of actions and/or adverse consequences that entailed participation, i.e., this is a kind of heroism when feeling duty is more important than high personal costs (Vilas and Sabucedo, 2012). In other words, a moral obligation that encompasses five components: (1) the sense of obligation itself; (2) autonomy; (3) personal satisfaction (if the behavior is consistent with the obligation); (4) discomfort (in case the behavior does not correspond to the obligation); (5) sacrifice (Sabucedo et al., 2018), which are important aspects of the individual differences of the protesters, motivates them to participate in collective action more than anything else.

### Limitations and further directions

Like all studies, the current is not without limitations. First, the surveyed study participants are not random probability samples of the populations of their countries of residence. While we controlled for many of the differences in our analyses, nonetheless, the generalizability of our findings is difficult to assess. However, as suggested by Stroebe et al. (2018) and Coppock et al. (2018), cross-cultural data are still useful even when samples are not fully representative. Stroebe et al. (2018) highlight the primacy of a robust theoretical framework over sample representativeness, indicating that meaningful findings can be derived even from less representative samples if they align with strong theoretical underpinnings. Coppock et al. (2018) further challenge the notion that only representative samples yield generalizable results, showing that non-representative samples can also provide consistent, valuable insights. This collective perspective underscores that the generalizability of research findings hinges more on theoretical and empirical robustness than on the demographic makeup of the sample, thereby supporting the relevance of our study despite its sampling limitations. In addition, many of the challenges inherent in non-representative samples would make cross-cultural *similarities* more *difficult* to discover. In our case, that makes our findings concerning the pancultural validity of many of our model’s conclusions even more impressive.

Another limitation of our study is that it did not consider the distinction between normative and non-normative collective action. Distinguishing between normative collective actions (i.e., those that conform to the norms of the existing social system, such as political participation or peaceful protest) and non-normative collective actions (i.e., those accompanied by, for example, violence), in some cases may require some specificity (Adam-Troian et al., 2021; see also, e.g., Tausch et al., 2011; Shuman et al., 2016). For example, national identification, among all other variables, positively predicted the intention to engage in normative collective action, but negatively predicted the intention to engage in non-normative collective action (see Stathi et al., 2019). These authors concluded that national identification is a factor that, on the one hand, motivates people to mobilize, but, on the other hand, prevents the negative and destructive side of collective action.

Corruption can refer to both (1) actions to obtain fair treatment (i.e., ‘need corruption,’ e.g., to get what is legally required) and (2) actions undertaken in order to obtain special illicit advantages that persist even in societies with well-established institutions of democratic accountability (i.e., ‘greed corruption,’ e.g., to get what is not legally allowed). In the case of need corruption, when individuals are forced to involve in corruption for the reason of limited access to public goods (e.g., education, healthcare), the motivation to participate in collective action may differ from greed corruption within which people also benefit to some extent (Bauhr, 2017). For instance, in the need condition, people are more likely to evaluate it as such and, in turn, protest against it. In other words, it is likely that each of these forms of corruption has its own motivational dynamics behind collective action and system justification, which might yield different forms of protests.

The question remains about the role of other macrosocial indicators, in which countries differ. For example, the implications of the level of social inequality in a society for collective action against corruption in a country, as well as its interaction with individual differences in supporting inequality, need to be explored. Indeed, the ideological endorsement of inequality at the individual level through social dominance orientation increases corrupt intent (see Vilanova et al., 2022). The role of higher-order factors, particularly during significant economic upheavals, is an area warranting exploration. Economic crises and soaring unemployment rates can often amplify or alter the effects of individual variables, leading to an upsurge in collective action. Thus, an interesting avenue for future research would be to examine the interplay between individual and societal-level variables in periods of economic stability and crisis, to gain a more nuanced understanding of the triggers for collective mobilization.

In a world where countries are becoming increasingly culturally diverse due to immigration, the effect of this heterogeneity on collective action is another vital area to explore. For instance, it has been observed that immigrant groups with strong national identification may exhibit less support for collective action (Mähönen and Jasinskaja-Lahti, 2015), potentially due to their additional ethnic identities, leading them to perceive national issues, such as corruption, as less personally relevant.

A separate body of literature suggests that individuals might tolerate corruption or organized crimes when the guilty party embodies the group’s values (e.g., Travaglino and Abrams, 2019). This might be particularly relevant in autocratic states where propaganda and various forms of control effectively color the state as embodying national values. In contrast, democratic states, due to their inherent pluralism, might be less effective in this aspect, potentially explaining the relatively small effects of national identity observed in our study. These complexities surrounding the context-specific acceptance of corruption offer a rich avenue for future research.

## Conclusion

The findings together demonstrate that while we can identify pancultural similarities, each context of collective action is nonetheless unique in some way. As it turns out, believing that they can change the situation is not always necessary, and the influence of such a belief is culturally constrained. However, there is nonetheless a thread across cultural contexts—a common view of the situation, an experience of dissatisfaction, deprivation, negative emotions (most often anger), and a feeling of moral obligation to participate in defending their position (despite the possible costs and negative consequences). Thus, in general our work reveals that while some variables can be considered as context-dependent, moral obligation in particular can be considered as a superior proximal predictor of collective action over perceived efficacy across the cultures we studied.

## Data availability statement

The original contributions presented in the study are included in the article/Supplementary material, further inquiries can be directed to the corresponding author.

## Ethics statement

Ethical approval was not required for the studies involving humans because this study was conducted in compliance with the ethical standards of COPE and APA. The procedure was in line with Russian regulations; as per university and national Russian regulations, no ethics clearance was required for this type of survey research (if it did not include medical data). The studies were conducted in accordance with the local legislation and institutional requirements. The participants provided their written informed consent to participate in this study.

## Author contributions

DG: Conceptualization, Data curation, Funding acquisition, Investigation, Methodology, Supervision, Writing – original draft. AG: Formal analysis, Project administration, Software, Validation, Visualization, Writing – original draft, Writing – review & editing. LC: Supervision, Writing – original draft, Writing – review & editing. AZ: Writing – original draft, Writing – review & editing. JS: Conceptualization, Methodology, Supervision, Writing – review & editing. MD: Conceptualization, Methodology, Writing – review & editing. AB: Conceptualization, Data curation, Methodology, Resources, Writing – original draft. KB: Supervision, Writing – review & editing.

## Footnotes

^1^We chose countries that embody distinct characteristics in three key dimensions: cultural orientation (individualistic vs. collectivistic), political regime (democratic vs. authoritarian), and economic status (developing vs. developed). This selection method ensures a comprehensive analysis across varied global contexts. Additionally, the choice of these countries aligns with the Inglehart-Welzel Cultural World Map, where each country represents a unique zone on the map. Notably, India is included as a significant outlier, offering a unique perspective from the West and South Asia zone.

^2^While acknowledging the potential for corruption in both democratic and autocratic systems, it is critical to emphasize the mechanisms in democracies that are designed to combat corruption. Democratic accountability, one of the key facets of democratic governance, allows for checks and balances on the actions of public officials. This transparency and accountability can often deter corrupt practices and provide means for prosecution when corruption is detected (Doorenspleet, 2019). Therefore, while not being immune to corruption, democracies possess inherent structures to counteract and reduce corruption, reinforcing the relevance of exploring this relationship in our study.

^3^In our statistical model, this suggests a reverse direction of the moderator’s effect; because—as noted above—higher CPI scores stand for less perception of corruption in the country.

^4^See https://www.worldbank.org/.

^5^See https://www.transparency.org/.
