# Supplementary material for: Collective action against corruption in Western and non-Western countries: cross-cultural implications of the Axiological-Identitary Collective Action Model
Source: Front Psychol. 2024 Mar 20;15:1269552. doi: 10.3389/fpsyg.2024.1269552 (PMC10987692; doi:10.3389/fpsyg.2024.1269552)
Supplement: Supplementary file 2 [file Table_2.DOCX]

| **English** | **Spanish** | **Russian** | **German** |
| --- | --- | --- | --- |
| **Moral obligation** | | | |
| *Please rate the following statements based on your beliefs about what obligations you personally have (in terms of how you should or should do so) in relation to the society in which you live.* | *Por favor, valore los siguientes enunciados en base a sus creencias acerca de sus obligaciones personales (en términos de cómo debería actuar o no) en relación con la sociedad en la que usted vive.* | *Пожалуйста, оцените следующие высказывания исходя из Ваших убеждений о том, какие обязательства имеете Вы лично по отношению к обществу, в котором Вы живёте (т.е. как Вы должны или не должны поступать).* | *Bitte bewerten Sie die folgenden Aussagen basierend auf Ihren Überzeugungen darüber, welche Verpflichtungen Sie persönlich in Bezug auf die Gesellschaft, in der Sie leben, haben (d.h. wie Sie dies tun sollten oder nicht).* |
| 1 = Strongly disagree; 2 = Disagree; 3 = Somewhat disagree; 4 = Neither agree or disagree; 5 = Somewhat agree; 6 = Agree; 7 = Strongly agree. | 1= totalmente en desacuerdo; 2= En desacuerdo; 3= Algo en desacuerdo; 4= Ni de acuerdo ni en desacuerdo; 5= Algo de acuerdo; 6= De acuerdo; 7= Totalmente de acuerdo | 1 = Абсолютно не согласен; 2 = Не согласен; 3 = Скорее не согласен; 4 = Не знаю, не уверен; 5 = Скорее согласен; 6 = Согласен; 7 = Абсолютно согласен. | 1 - stimmt überhaupt nicht zu; 2 - stimmt nicht zu; 3 - stimmt eher nicht zu; 4 - weiß nicht; 5 - stimmt eher zu; 6 - stimmt zu; 7 - stimmt voll und ganz zu |
| To mobilize against corruption constitutes a moral obligation to oneself. | Movilizarse contra en contra de la corrupción constituye una obligación moral para con uno mismo. | Противодействие коррупции в России является для меня моральным обязательством перед самим собой. | Mobilisierung gegen die Korruption stellt eine moralische Verpflichtung für mich dar. |
| To mobilize against corruption would make me feel proud of myself. | Movilizarme en contra de la corrupción me haría sentir orgulloso/a de mí mismo/a. | Мой личный вклад в противодействие коррупции в России заставил бы меня гордиться собой. | Gegen die Korruption zu mobilisieren, würde mich stolz auf mich machen. |
| To not mobilize against corruption would make me feel guilty. | No movilizarme en contra de la corrupción me haría sentir culpable. | Отсутствие противодействия коррупции в России с моей стороны, заставило бы меня чувствовать вину. | Wenn ich nicht gegen die Korruption mobilisierte, würde ich mich schuldig fühlen. |
| I feel morally obliged to mobilize against corruption even when that means confronting people that are close to me. | Me siento moralmente obligado/a a movilizarme en contra de la corrupción incluso si ello supone enfrentarme a personas cercanas a mí. | Я чувствую, что морально обязан мобилизоваться против коррупции в России, даже если это как-то связано с неким противостоянием близким мне людям. | Ich fühle mich moralisch verpflichtet, gegen die Korruption zu mobilisieren, auch wenn das bedeutet, Menschen zu konfrontieren, die mir nahe stehen. |
| No matter what anyone thinks, I feel morally obliged to participate in demonstrations against corruption. | Independientemente de lo que los demás piensen, me siento moralmente obligado/a a participar en movilizaciones en contra de la corrupción. | Не важно, что подумают другие, я чувствую моральную обязанность участвовать в демонстрациях против коррупции в России. | Egal, was irgendjemand denkt, fühle ich mich moralisch verpflichtet, an Demonstrationen gegen die Korruption teilzunehmen. |
| **General system justification** | | | |
| *Think about the current situation in the country, how much do you agree with the following statements:* | *Piense acerca de la situación actual del país, en qué medida está de acuerdo con los siguientes enunciados:* | *Подумайте, о сегодняшней ситуации в России, насколько Вы согласны со следующими утверждениями:* | *Denken Sie an die aktuelle Situation in Deutschland, wie sehr stimmen Sie den folgenden Aussagen zu:* |
| 1 = Strongly disagree; 2 = Disagree; 3 = Somewhat disagree; 4 = Neither agree or disagree; 5 = Somewhat agree; 6 = Agree; 7 = Strongly agree. | 1= totalmente en desacuerdo; 2= En desacuerdo; 3= Algo en desacuerdo; 4= Ni de acuerdo ni en desacuerdo; 5= Algo de acuerdo; 6= De acuerdo; 7= Totalmente de acuerdo | 1 = Абсолютно не согласен; 2 = Не согласен; 3 = Скорее не согласен; 4 = Не знаю, не уверен; 5 = Скорее согласен; 6 = Согласен; 7 = Абсолютно согласен. | 1 - stimmt überhaupt nicht zu; 2 - stimmt nicht zu; 3 - stimmt eher nicht zu; 4 - weiß nicht; 5 - stimmt eher zu; 6 - stimmt zu; 7 - stimmt voll und ganz zu |
| In general, you find society to be fair. | En general, encuentro que la sociedad es justa. | В целом, я нахожу наше сегодняшнее общество справедливым. | Im allgemeinen finde ich die Gesellschaft fair. |
| In general, the American system operates as it should. | En general, el sistema político español funciona como debería. | В целом, российская политическая система работает так, как должна. | Im allgemeinen funktioniert das politische System Deutschlands, wie es sollte. |
| American society needs to be radically restructured. | La sociedad española necesita ser radicalmente reestructurada. | Сегодняшнему российскому обществу нужна радикальная перестройка. | Die deutsche Gesellschaft muss radikal umstrukturiert werden. |
| The United States is the best country in the world to live in. | España es el mejor país del mundo en el que vivir. | Для меня, наше государство — лучшее место в мире для жизни. | Deutschland ist das beste Land der Welt, um darin zu leben. |
| Most policies serve the greater good. | La mayoría de las medidas políticas favorecen el bien común. | Проводимая в нашей стране политика служит улучшению нашей жизни. | Die meisten politischen Entscheidungen dienen dem Wohle aller. |
| Everyone has a fair shot at wealth and happiness. | Todo el mundo tiene una oportunidad legítima de alcanzar riqueza y felicidad. | В России каждый имеет честные шансы для благополучия и счастья. | Alle Menschen haben faire Chancen, Glück und Wohlstand zu erlangen. |
| Our society is getting worse every year. | Nuestra sociedad empeora día a día. | В России становится хуже год от года. | Unsere Gesellschaft verschlechtert sich Jahr für Jahr. |
| Society is set up so that people usually get what they deserve. | La sociedad está articulada de modo que la gente habitualmente consigue lo que merece. | Российское общество устроено таким образом, что люди обычно получают то, что они заслуживают. | In unserer Gesellschaft bekommen Menschen für gewöhnlich, was sie verdienen. |
| **National identification** | | | |
| *People can perceive themselves very differently. The following statements relate to how you perceive yourself.* | *La gente se percibe a sí misma de modos muy distintos. Los siguientes enunciados se relacionan con cómo usted mismo se percibe.* | *Ниже вопросы о том, насколько Вы себя отождествляйте с россиянами (гражданская принадлежность). Оцените, насколько Вы согласны со следующими утверждениями, следуя 7 балльной шкале:* | *Die Menschen können sich selbst ganz unterschiedlich wahrnehmen. Die nachfolgenden Fragen beziehen sich darauf, wie Sie sich in diesem Sinne selbst wahrnehmen.* |
| 1 = Strongly disagree; 2 = Disagree; 3 = Somewhat disagree; 4 = Neither agree or disagree; 5 = Somewhat agree; 6 = Agree; 7 = Strongly agree. | 1= totalmente en desacuerdo; 2= En desacuerdo; 3= Algo en desacuerdo; 4= Ni de acuerdo ni en desacuerdo; 5= Algo de acuerdo; 6= De acuerdo; 7= Totalmente de acuerdo | 1 = Абсолютно не согласен; 2 = Не согласен; 3 = Скорее не согласен; 4 = Не знаю, не уверен; 5 = Скорее согласен; 6 = Согласен; 7 = Абсолютно согласен. | 1 - stimmt überhaupt nicht zu; 2 - stimmt nicht zu; 3- stimmt eher nicht zu; 4 - weiß nicht; 5 - stimmt eher zu; 6 - stimmt zu; 7 - stimmt voll und ganz zu |
| I consider myself a [...] ([country] citizen). | Me considero a mí mismo español /ciudadano español. | Я считаю себя россиянином/россиянкой (гражданином России). | Ich nehme mich als Staatsbürger / Staatsbürgerin Deutschlands wahr. |
| I feel like a part of [...] society. | Me siento parte de la sociedad española. | Я чувствую себя частью российского общества. | Ich nehme mich als Teil der deutsche Gesellschaft wahr. |
| **Perceived efficacy** | | | |
| *Please indicate how much you agree with each of the following statements.* | *Por favor, indique en qué medida usted está de acuerdo con los siguientes enunciados.* | *Пожалуйста, укажите в какой степени Вы согласны или не согласны с высказываниями ниже.* | *Geben Sie bitte an, wie sehr Sie mit jeder der nachfolgenden Aussagen einverstanden sind.* |
| 1 = Strongly disagree; 2 = Disagree; 3 = Somewhat disagree; 4 = Neither agree or disagree; 5 = Somewhat agree; 6 = Agree; 7 = Strongly agree. | 1= totalmente en desacuerdo; 2= En desacuerdo; 3= Algo en desacuerdo; 4= Ni de acuerdo ni en desacuerdo; 5= Algo de acuerdo; 6= De acuerdo; 7= Totalmente de acuerdo | 1 = Абсолютно не согласен; 2 = Не согласен; 3 = Скорее не согласен; 4 = Не знаю, не уверен; 5 = Скорее согласен; 6 = Согласен; 7 = Абсолютно согласен. | 1 - stimmt überhaupt nicht zu; 2 - stimmt nicht zu; 3 - stimmt eher nicht zu; 4 - weiß nicht; 5 - stimmt eher zu; 6 - stimmt zu; 7 - stimmt voll und ganz zu |
| I can contribute to the collective actions that affect the government. | Puedo contribuir en acciones colectivas (movilizaciones) que afectan las decisiones del gobierno. | Я могу внести свой вклад в коллективные действия, которые влияют на правительство. | Ich kann zu den kollektiven Aktionen beitragen, die die Regierung betrifft. |
| I can contribute to the collective actions that affect society as a whole. | Puedo contribuir en acciones colectivas (mobilizaciones) que afectan a la sociedad en su conjunto. | Я могу внести свой вклад в коллективные действия, которые влияют на общество в целом. | Ich kann zu den kollektiven Aktionen beitragen, die die Gesellschaft als Ganzes betreffen. |
| **System-based anger** | | | |
| *Please indicate how much you agree with each of the following statements.* | *Por favor, indique en qué medida usted está de acuerdo con los siguientes enunciados.* | *Пожалуйста, укажите в какой степени Вы согласны или не согласны с высказываниями ниже.* | *Geben Sie bitte an, wie sehr Sie mit jeder der nachfolgenden Aussagen einverstanden sind.* |
| 1 = Strongly disagree; 2 = Disagree; 3 = Somewhat disagree; 4 = Neither agree or disagree; 5 = Somewhat agree; 6 = Agree; 7 = Strongly agree. | 1= totalmente en desacuerdo; 2= En desacuerdo; 3= Algo en desacuerdo; 4= Ni de acuerdo ni en desacuerdo; 5= Algo de acuerdo; 6= De acuerdo; 7= Totalmente de acuerdo | 1 = Абсолютно не согласен; 2 = Не согласен; 3 = Скорее не согласен; 4 = Не знаю, не уверен; 5 = Скорее согласен; 6 = Согласен; 7 = Абсолютно согласен. | 1 - stimmt überhaupt nicht zu; 2 - stimmt nicht zu; 3 - stimmt eher nicht zu; 4 - weiß nicht; 5 - stimmt eher zu; 6 - stimmt zu; 7 - stimmt voll und ganz zu |
| I am angry because of what political path [country] is currently on. | Me produce enfado el curso que está tomando la política en España actualmente. | Я злюсь из-за того, по какому политическому пути сейчас идёт Россия. | Ich ärgere mich darüber, auf welchem politischen Weg Deutschland gerade steht. |
| I feel anger when I think about the current state of affairs in [country]. | Siento enfado cuando pienso acerca del estado de las cosas en España | Я испытываю гнев, когда думаю о текущем положении дел в России. | Ich fühle Wut, wenn ich an den aktuellen Stand der Dinge in Deutschland denke. |
| **Collective action** | | | |
| *Please indicate how much you agree with each of the following statements.* | *Por favor, indique en qué medida usted está de acuerdo con cada uno de los siguientes enunciados.* | *Пожалуйста, укажите в какой степени Вы согласны или не согласны с высказываниями ниже.* | *Geben Sie bitte an, wie sehr Sie mit jeder der nachfolgenden Aussagen einverstanden sind.* |
| 1 = Strongly disagree; 2 = Disagree; 3 = Somewhat disagree; 4 = Neither agree or disagree; 5 = Somewhat agree; 6 = Agree; 7 = Strongly agree. | 1= totalmente en desacuerdo; 2= En desacuerdo; 3= Algo en desacuerdo; 4= Ni de acuerdo ni en desacuerdo; 5= Algo de acuerdo; 6= De acuerdo; 7= Totalmente de acuerdo | 1 = Абсолютно не согласен; 2 = Не согласен; 3 = Скорее не согласен; 4 = Не знаю, не уверен; 5 = Скорее согласен; 6 = Согласен; 7 = Абсолютно согласен. | 1 - stimmt überhaupt nicht zu; 2 - stimmt nicht zu; 3 - stimmt eher nicht zu; 4 - weiß nicht; 5 - stimmt eher zu; 6 - stimmt zu; 7 - stimmt voll und ganz zu |
| I am ready to participate in the action against corruption in [country]. | Estoy dispuesto/a a participar en acciones en contra de la corrupción en España | Я готов принять участие в акции против коррупции в России. | Ich bin bereit, an der Aktion gegen die Korruption in Deutschland teilzunehmen. |
| I am ready to support protests (e.g., rallies, marches) against corruption in [country]. | Estoy dispuesto/a a apoyar protestas (p.ej. manifestaciones, marchas…) en contra de la corrupción en España | Я готов поддержать протестные действия (напр., митинги, шествия) против коррупции в России. | Ich bin bereit, Protestaktionen (zB Kundgebungen, Märsche) gegen Korruption in Deutschland zu unterstützen. |
| **Gender** | | | |
| Gender | Género (Mujer-Woman; Hombre-Man; No me identifico con ninguno de los anteriores- I don't identify with any of the previous) | Пожалуйста, укажите Ваш пол. | Sie sind |
| **Age** | | | |
| How old are you? | Por favor, indique su edad | Сколько Вам лет? | Wie alt sind Sie? |
| **Student status** | | | |
| Are you currently a student? | En este momento, ¿es usted estudiante? | На данный момент Вы являетесь студентом/студенткой? | Sind Sie Student/in? |

Source: *Collective Action against Corruption in Western and non-Western Countries: Cross-Cultural Implications of the Axiological-Identitary Collective Action Model*
